# Transitioning of Patients from Direct-Acting Oral Anticoagulant to Heparin: Impact on Laboratory Testing

**DOI:** 10.1055/a-2734-2293

**Published:** 2025-11-13

**Authors:** Deepa J. Arachchillage, Saravanan Vinayagam, Annie Parsons, Nilanthi Karawitage, Mike Laffan

**Affiliations:** 1 Department of Immunology and Inflammation, Centre for Haematology, Imperial College London, United Kingdom; 2 Department of Haematology, Imperial College Healthcare NHS Trust, London, United Kingdom; 3 North West London Pathology, Imperial College Healthcare NHS Trust, London, United Kingdom

**Keywords:** direct oral anticoagulants, heparin, anti-Xa, activated partial thromboplastin time, monitoring

## Abstract

Direct oral anticoagulants (DOACs), including direct thrombin inhibitors (dabigatran) and direct factor Xa inhibitors (apixaban, rivaroxaban, edoxaban), have transformed anticoagulant management in recent years due to their predictable pharmacodynamics, rapid onset of action, and fixed dosing without the need for routine laboratory monitoring. Unfractionated heparin (UFH) remains the anticoagulant of choice for patients who are acutely unwell and treated in intensive care units due to its short half-life, reversibility, ease of dose titration, and nonrenal dependent excretion. It is therefore not uncommon for an individual's anticoagulation management to require rapid changing from DOAC to UFH. Due to UFH's complex pharmacokinetics, including nonspecific binding to acute phase proteins and dose-dependent clearance, careful laboratory monitoring, generally with activated partial thromboplastin time (APTT) or anti-factor Xa (anti-Xa) activity, is necessary. When transitioning from a DOAC to UFH, overlapping pharmacologic effects can significantly interfere with coagulation assays, particularly if residual DOAC levels persist at the time UFH is initiated. DOACs can prolong the APTT and elevate anti-Xa activity, leading to overestimation of UFH activity, inappropriate dose adjustments, and increased risk of bleeding or thromboembolic events. Here, we examine the laboratory implications of transitioning from DOAC therapy to UFH, with a focus on the performance and interpretation of APTT and anti-Xa assays in the presence of residual DOAC levels and how to overcome the interference of DOAC in UFH monitoring. We suggest an algorithm to follow during this transition.

Direct oral anticoagulants (DOACs), which include direct thrombin inhibitors (dabigatran) and direct factor Xa inhibitors (apixaban, rivaroxaban, edoxaban), have transformed anticoagulant management in recent years due to their predictable pharmacodynamics, rapid onset of action, and fixed dosing without the need for routine laboratory monitoring. 1 2 DOAC have become the anticoagulant of choice for many indications, including treatment and prevention of venous thromboembolism (VTE) and prevention of stroke and systemic embolization in patients with atrial fibrillation, replacing vitamin K antagonists (VKA). Their adoption has significantly reduced the burden of frequent blood tests for international normalized ratio monitoring and dose adjustment required for VKA.

In contrast, unfractionated heparin (UFH) remains the anticoagulant of choice for patients who are acutely unwell and treated in intensive care units due to its short half-life, reversibility, ease of dose titration, and nonrenal dependent excretion. However, its narrow therapeutic window and complex pharmacokinetics, including nonspecific binding to acute phase proteins and dose-dependent clearance, necessitate careful laboratory monitoring, traditionally via the activated partial thromboplastin time (APTT) or anti-factor Xa (anti-Xa) activity. The accuracy of these assays is critical for safe and effective dosing. When transitioning from a DOAC to UFH, overlapping pharmacologic effects can significantly interfere with coagulation assays, particularly if residual DOAC is present at the time UFH is initiated. DOACs can prolong the APTT and elevate anti-Xa activity, leading to overestimation of UFH activity, inappropriate dose adjustments, and increased risk of bleeding or thromboembolic events. Low molecular weight heparin (LMWH), which is a fractionated form of UFH, is more commonly used in hospitalized patients, especially when there is reasonable renal function (generally creatinine clearance > 30 mL/minute), due to its subcutaneous injection, lack of need for regular monitoring and several other advantages over UFH which are discussed in detail later in this paper.

Despite the clinical importance of this transitional period, data are limited on how residual DOAC concentrations influence the laboratory markers used to monitor UFH. Current guidelines provide general recommendations but lack detailed strategies to mitigate assay interference or to guide the timing of transition. This gap in evidence is particularly problematic in high-risk populations, such as the critically ill or those requiring urgent invasive interventions.

The aim of this study is to examine the laboratory implications of transitioning from DOAC therapy to UFH, with a focus on the performance and interpretation of APTT and anti-Xa assays in the presence of residual DOAC levels, and how to overcome the interference of DOAC in UFH monitoring with evidence. Finally, we provide an algorithm to follow if a patient is transitioning from a DOAC to UFH in clinical practice.

## Material and Methods

This study was approved by the Human Research Authority and Health and Care Research Wales (reference: 22/PR/145). We used the existing data from the literature and the data generated from UFH spiked into pooled normal plasma and samples obtained from patients treated with UFH or DOACs. Pooled normal plasma was prepared by pooling the plasma processed from blood obtained from 20 healthy controls not on any antithrombotic treatment following informed consent. Residual plasma samples from patients on UFH or DOAC were taken when they had routine coagulation testing. Both healthy controls and patients' samples were taken into 0.105 M citrate Vacutainers (Becton Dickinson, Plymouth, United Kingdom). Plasma was prepared within 1 hour by double centrifugation at 2,000 g for 15 minutes and stored at −70°C until analysis. 3

APTT was measured using six different APTT reagents on the Stago STA R Max2 (Stago, United Kingdom) analyzer. Heparin anti-Xa and drug-specific direct factor Xa levels (STA-Liquid Anti-Xa, Stago, United Kingdom) were measured by the same analyzer. DOAC-Stop (Haematex Products, Australia) was used to remove the DOAC from patients' samples. Here, a DOAC stop mini-tab is added to 1 mL of citrated plasma and gently mixed until dispersed. After further mixing for 5 to 10 minutes, the sample is centrifuged for 5 minutes at 6,000 rpm or 2,000 g. The supernatant plasma is removed gently and tested for DOAC anti-Xa activity using drug-specific calibrators (STA-Liquid Anti-Xa Stago, United Kingdom).

### Statistical Analysis

Two groups were compared by either an unpaired *t* -test or Mann–Whitney *U* test, depending on the distribution of the data. The normality of data distribution was assessed with the Shapiro–Wilk test. Correlation between APTT versus heparin anti-Xa levels was assessed using Spearman correlation. Statistical significance was defined as *p* < 0.05 with *p* -values adjusted by Bonferroni correction.

## Results and Discussion

### DOACs and Coagulation Assays

DOACs interfere with most clot-based coagulation assays, including routine coagulation screen tests, prothrombin time (PT), and APTT. 4 5 Due to the variable sensitivity of the thromboplastin and APTT reagents used to assess the PT and APTT, the same level of DOAC can produce a range of PT and APTT values in different laboratories, and these tests are unsuitable for estimating DOAC exposure or even to exclude clinically relevant plasma DOAC levels. 6 7 8 The presence of DOAC in patient samples will interfere with Protein C, Protein S, or antithrombin (AT) functional assays. Protein C clot-based assays contain a heparin neutralizer, negating the impact of heparin, and the Protein C chromogenic assay is considered not to be impacted by DOACs. 9 In addition, Protein S activity assays are not recommended as initial screening tests, with the protein S free antigen assay being preferred, and this would not be impacted by either heparin or DOACs. Furthermore, the presence of DOAC causes underestimation of coagulation proteins such as factor VIII, IX X if measured by functional assays. 4 10

Although routine monitoring is not required, there are certain situations in which assessing DOAC levels may be indicated, as suggested by the recently published British Society of Haematology (BSH) guidelines on measurement of heparin, direct oral anticoagulants, and other noncoumarin anticoagulants and their effects on haemostasis assays. Patients with renal impairment, concomitant use of an interacting drugs, at extremes of body weight (e.g., <50 or >120 kg), patients presenting severe, acute haemorrhage, following suspected overdose, prior to an invasive procedure with a high bleeding risk, when the time from the last dose is not known, patients with gastrointestinal abnormalities causing impaired absorption, to assess the compliance of the patients, and in case of use of nonstandard doses of DOAC are the suggested indications for the testing of DOAC levels. 9

Direct factor Xa inhibitors, including rivaroxaban, apixaban, and edoxaban, are measured in routine clinical practice using a chromogenic substrate Factor Xa assay with drug-specific calibrators, while the anticoagulant activity of dabigatran is assessed using different assays whose endpoint is dependent on thrombin function.

### Unfractionated Heparin

UFH is a sulphated polysaccharide with a mean molecular weight of 15,000 Da (range: 3,000–30,000), discovered in the 1930s. UFH inactivates thrombin and factor Xa through an AT-dependent mechanism. Heparin binds to AT through a high-affinity pentasaccharide, which is present on approximately 30% of heparin molecules. Due to its short half-life, availability of antidote (protamine sulfate), wide range of therapeutic effect and excretion which is not renal dependent, it is the anticoagulant of choice for patients undergoing cardiac surgery, patients supported by extracorporeal membrane oxygenation (ECMO), critically unwell patients with a high risk of bleeding and renal failure and situations where rapid change of anticoagulation is required. 11 As heparin is a highly negatively charged molecule, it has the propensity to bind to positively charged proteins and surfaces, many of which (e.g., VWF and fibrinogen) are increased when patients are acutely unwell with infection/inflammation requiring intensive care treatment. This leads to a variable anticoagulant response, not directly proportional to the dose of heparin, and the need to monitor the anticoagulant effect. 12 As only one-third of heparin molecules have the anticoagulant pentasaccharide, functional assays are more valuable than physical measurement of heparin concentration. The most commonly used laboratory assays include APTT for therapeutic anticoagulation and the activated clotting time (ACT) for high concentrations in cardiopulmonary bypass (CPB). Both of these are subject to multiple confounders, and heparin anti-Xa activity using a heparin-specific calibrator is a more accurate functional measure of its anticoagulant effect. Although UFH also has a significant anti-FIIa effect, this is not often measured in clinical practice. Further complicating the matter, therapeutic targets for UFH are poorly defined. The currently used target 1.5 to 2.5 APTT ratio (APTTR) is derived from post hoc analysis of a descriptive study published in 1972, which included 234 patients treated with continuous intravenous infusions of UFH, showing that an APTTR of 1.5 to 2.5 is associated with a lower risk of recurrent VTE and also reduced thrombus extension in rabbits. 13 Subsequently, it was shown that this APTTR ratio corresponded to 0.2 to 0.4 IU/mL by protamine neutralization and 0.3 to 0.7 IU/mL by anti-Xa assay. However, depending on the APTT reagents used in different laboratories, the APTTR ranges corresponding to heparin anti-Xa 0.3 to 0.7 IU/mL vary from 1.6 to 2.7 to 3.7 to 6.2 IU/mL. 14 Therefore, local laboratories should determine their own APTTR range equivalent to heparin anti-Xa of 0.3 to 0.7 IU/mL. 9

APTT and heparin anti-Xa show a linear relation when UFH is spiked into pooled normal plasma, as the variation here is only the dose of UFH ( Fig. 1A ). This linear response is not unexpected; however, the APTT prolongation is significantly greater than that observed in heparinized patient samples due to lack of nonspecific binding and other factors described elsewhere in this manuscript. In contrast, when samples taken from patients on UFH are analyzed, there is only moderate correlation between heparin anti-Xa and APTT due to variation arising from patients ( Fig. 1B ).

**Fig. 1:**
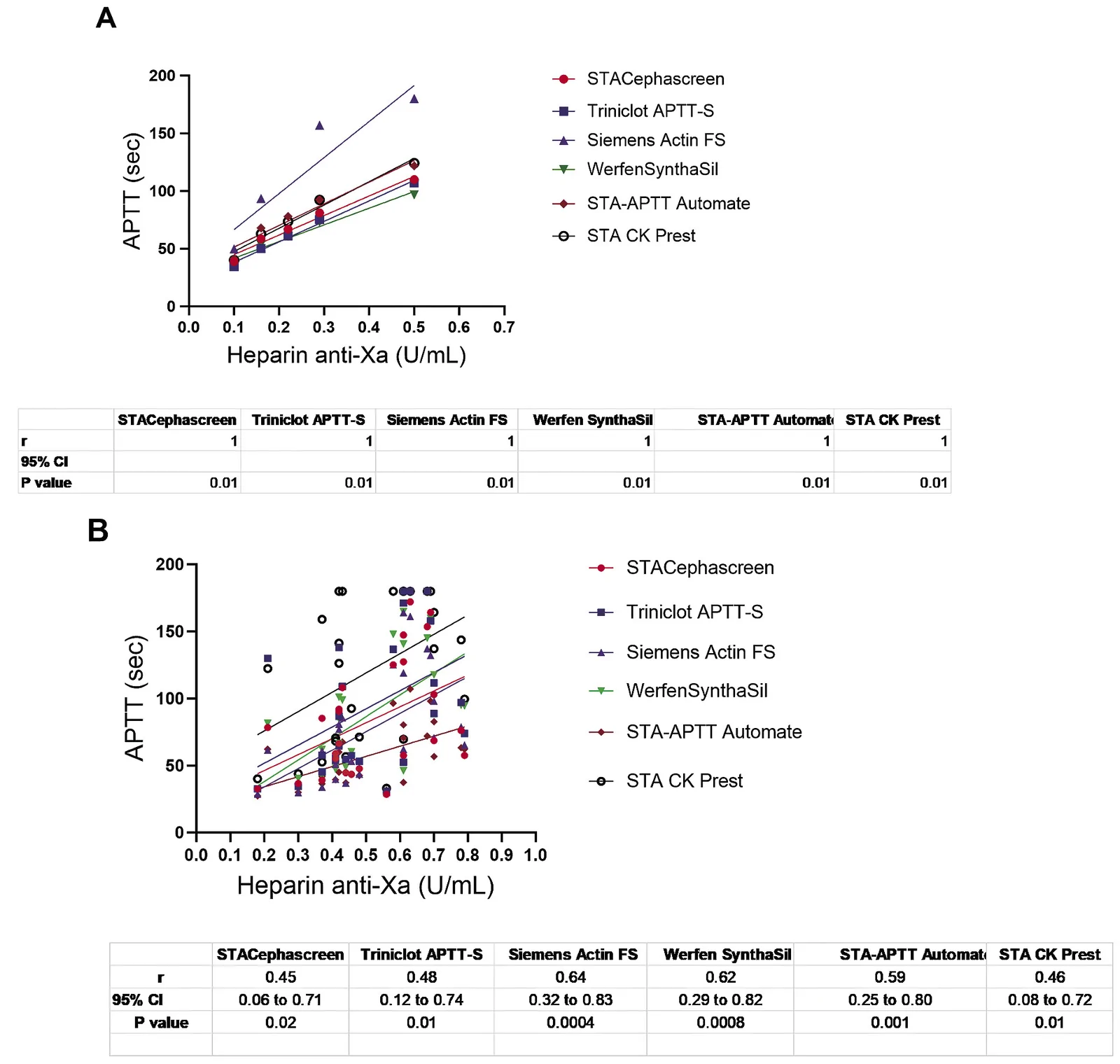
Heparin anti-Xa versus APTT in pooled normal plasma spiked with unfractionated heparin shows excellent correlation between the two assays ( **A** ), and heparin anti-Xa versus APTT in patients on unfractionated heparin shows moderate correlation between the two assays ( **B** ).

### Low Molecular Weight Heparin

LMWH is generated from UFH via chemical or enzymatic degradation with a corresponding reduction in anti-thrombin activity relative to anti-factor Xa activity. LMWH has one-third the molecular weight of UFH (4,000–5,000 Da). It has less nonspecific binding to plasma proteins and cells, hence a more predictable anticoagulant effect. Therefore, monitoring is required only in certain situations, such as renal failure, bleeding, extreme body weight, mechanical heart valves, or during pregnancy. UFH has been largely replaced by LMWH due to its more reliable dose response, without the need for monitoring, longer half-life, subcutaneous injection rather than continuous IV infusion, and lower incidence of heparin-induced thrombocytopenia and osteopenia. 11 It has also been suggested that FXa is a better antithrombotic target than FIIa, and although this is less clear, LMWH anti-Xa activity is not well correlated with the risk of bleeding or thrombosis, and routine monitoring does not improve clinical outcomes. Unlike UFH, where clearance of the drug is mainly through the reticular endothelial system, elimination of LMWH is mainly through a nonsaturable renal route. Hence, monitoring LMWH anti-FXa activity may offer reassurance for minimizing the risk of bleeding in patients with severe renal dysfunction. The target LMWH anti-FXa activity levels have not been clinically validated, and there is no standardized method for adjusting doses based on LMWH anti-FXa activity. However, some suggest that peak FXa levels between 1.0 to 2.0 and 0.6 to 1.0 IU/mL in samples taken 3 to 4 hours from the last dose of the drug in steady state are appropriate for once daily and twice daily regimens, respectively. 15 16 17 18 Depending on the type of LMWH and the APTT reagent used, the APTT may be slightly prolonged with LMWH in a dose-dependent manner. 19 Thrombin time (TT) is not expected to be prolonged at therapeutic concentrations of LMWH, but this varies depending on the reagents used and type of LMWH; for example, tinzaparin is reported to have a higher factor IIa affinity. 9 20

## Methods Used in Monitoring UFH and the Effect of DOAC on these Assays

1. APTT: it is commonly used to monitor UFH, although it can be confounded by preanalytical and analytical variables contributing to inaccurate assessment of the anticoagulant effect of heparin. DOACs, particularly dabigatran and direct factor Xa inhibitors, can also prolong the APTT to a variable degree, especially rivaroxaban, depending on the APTT reagent used, with Actin FS/FSL (Siemens, Germany) reagents showing the highest sensitivity to rivaroxaban. 9 21 This could possibly be due to the use of ellagic acid as the activator for these reagents. 22 Although the effect of UFH on PT is minimal due to the presence of heparin-neutralizing agents in PT reagents and not used in the monitoring of UFH, the PT can be prolonged to variable degrees in the presence of direct factor Xa inhibitors. The PT is also less sensitive to apixaban than rivaroxaban up to a drug concentration of 200 ng/mL, with considerable variation seen depending on thromboplastin, with IL Recombiplastin (Werfen, California) proving the most and Thromborel S (Siemens, Germany) the least sensitive to both apixaban and rivaroxaban. 9 21
In patients whose anticoagulation is switching from DOAC to heparin, confusion may arise because the APTT may be prolonged due to both agents, making it difficult to interpret the degree of heparin anticoagulation. Although some experts suggest using an APTT assay for anticoagulation monitoring during this transition, 23 preanalytical and analytical variables and confounding factors make the APTT not ideal to monitor UFH. The pharmacokinetic properties of DOACs versus UFH are summarized in Table 1 .
2. ACT: is a point-of-care test used to monitor the anticoagulant effect of UFH during procedures requiring high-dose heparin, such as CPB, percutaneous coronary interventions, and patients supported by ECMO. The ACT is influenced by hypothermia, haemodilution, platelet count, and function, which are relevant to overall in vivo coagulation but lead to dissociation from the anticoagulant effect of UFH. DOACs have limited and variable effects on ACT, and any prolongation of ACT is modest, dose-dependent, and inconsistent between patients. An in vitro study supported that dabigatran interferes more than oral direct factor Xa inhibitors with the ACT. 24 Therefore, there can be a variable to no effect on ACT prolongation depending on the type and level of DOAC in a patient transitioning from a DOAC to UFH.
3. Heparin anti-Xa: as anti-Xa assays are used to measure both heparin and DOAC anticoagulant activity, the interference of direct FXa inhibitors can lead to overestimation of heparin anti-Xa levels, suggesting a higher-than-actual heparin activity in the blood despite using heparin-specific calibrators. 25 Although heparin can also lead to overestimation of DOAC-specific anti-Xa level, testing the DOAC level immediately after switching from heparin to DOAC is not required.

**Table 1:** Pharmacokinetic properties of direct-acting oral anticoagulants versus unfractionated heparin

Pharmacokinetic property	DOACs (rivaroxaban, apixaban, edoxaban, dabigatran)	Unfractionated heparin (UFH)
Route of administration	Oral	Intravenous (IV) or subcutaneous (SC)
Onset of action	1–4 h	Immediate (IV), 20–60 min (SC)
Half-life	5–17 h (drug-dependent)	30–90 min (dose-dependent)
Bioavailability	Moderate to high (e.g., rivaroxaban approximately 80–100%)	Poor 30–70% after SC injection
Protein binding	High (esp. rivaroxaban, apixaban)	Moderate (approximately 50–70%)
Metabolism	Hepatic (CYP3A4, P-gp, or esterases)	Minimal hepatic metabolism
Renal clearance	Moderate to high (esp. dabigatran approximately 80%)	Minor (mainly hepatic/RES)
Monitoring required	Not routine (except in special populations)	Yes (aPTT or heparin anti-Xa)
Dose individualization	Fixed dosing (weight- and renal function-adjusted)	Highly individualized
Interindividual variability	Low	High (due to nonspecific binding)
Drug–drug interactions	Moderate (CYP3A4, P-gp interactions)	Fewer significant interactions
Reversal agent	Available (e.g., idarucizumab, andexanet alfa)	Protamine sulfate

Supratherapeutic initial anti-Xa activity was reported in heparinized patients treated with oral FXa inhibitors within the previous 72 hours/1 week, even when the DOAC was undetectable (below the lower limit of quantitation) or at clinically insignificant levels (<30 ng/mL), according to reagent and calibration. 26 27 28 Heparin anti-Xa methods have variable sensitivity to DOACs, with the Stago heparin anti-Xa assay showing the highest sensitivity to rivaroxaban. 29 30 Measurement of anti-FIIa activity can avoid interference with UFH monitoring in patients with recent apixaban or rivaroxaban use, but this test is not widely used and is affected by dabigatran or argatroban. 31

Proposed suggestions to deal with interference of oral FXa inhibitors on heparin anti-Xa assay from the ISTH SSC subcommittee on control of anticoagulation are summarized in Table 2 . 27

**Table 2:** ISTH SSC subcommittee on control of anticoagulation proposed suggestions for dealing with interference of direct oral FXa inhibitors on heparin anti-Xa assay

• Physicians should inform the laboratory about recent (<1 wk) oral FXa inhibitor treatment when requesting APTT or anti-Xa for UFH monitoring
• Baseline UFH-calibrated anti-Xa activity test before UFH initiation should be considered to determine whether oral FXa inhibitor interference is present
• Unexpectedly high UFH-calibrated anti-Xa measured just after UFH introduction should prompt suspicion of interference with oral FXa inhibitors
• DOAC removal systems, once locally validated for use to isolate specific drug anti-Xa activity, can be used for UFH anti-Xa monitoring when UFH therapy is initiated after recent discontinuation of oral FXa inhibitors and until the interference is no longer present
• Laboratory and clinical stakeholders should create a written institutional protocol to detect and address DOAC interference on UFH monitoring

Source: Adapted with permission from Gendron et al. 27

### Thrombin Time, Diluted Thrombin Time, and Ecarin Clotting Time

TT, dilute thrombin time (dTT), and the ecarin clotting time (ECT) or ecarin chromogenic assay (ECA) can be used to assess direct thrombin inhibitor effect either qualitatively or quantitatively. The TT is useful for detecting the presence of dabigatran, but its extreme sensitivity limits its clinical utility for quantifying drug levels. dTT and the ECT or ECA have the ability to quantify dabigatran since the assays are less sensitive compared with TT and can be calibrated to measure the levels. The dTT involves diluting patient plasma and adding a standardized amount of thrombin to generate a clotting time that correlates linearly with dabigatran concentration. It is less susceptible to saturation than the unmodified TT and can be calibrated to estimate dabigatran levels with reasonable accuracy. These assays are highly specific for direct thrombin inhibitors and are unaffected by factor Xa inhibitors or other anticoagulants, making them especially valuable during transition to UFH. In clinical practice, ECT and dTT may help determine whether it is safe to initiate heparin therapy by confirming the absence or minimal presence of dabigatran activity. However, these assays are not widely available outside of specialized laboratories and are not routinely used in many institutions. Their limited accessibility underscores the need for cautious interpretation of standard coagulation tests and careful consideration of drug pharmacokinetics when managing dabigatran-to-UFH transitions. TT is not sensitive to the presence of direct factor Xa inhibitors. 21 The APTT has limited value in detecting dabigatran. Its response to dabigatran is nonlinear and is poor at high levels of the drug. The PT is not useful for the detection or quantification of dabigatran as its sensitivity is low.

## DOAC Stop or Removal Test can Be Used to Eliminate the Effect of DOAC on Patient Samples

An ISTH SSC subcommittee recommended that DOAC removal systems, once locally validated for use to isolate specific drug anti-Xa activity, can be used for UFH anti-Xa monitoring when UFH therapy is initiated after recent discontinuation of oral FXa inhibitors and until the interference is no longer present. Data presented in Fig. 2A, B demonstrate that in samples taken from patients on rivaroxaban or apixaban, treatment with DOAC stop (Haematex, Australia) can eliminate the effects of DOACs, demonstrating undetectable levels of rivaroxaban or apixaban as measured against drug-specific anti-Xa calibrators. As shown in Fig. 2A, B , both apixaban and rivaroxaban were reduced below the lower limits of detection (25 ng/mL for rivaroxaban and 23 ng/mL for apixaban, respectively), demonstrating that DOAC-stop can eliminate the effect of DOAC. In keeping with the findings from our results, previous studies also showed that DOAC removal 28 32 may be used to eliminate the effects of DOAC inference on heparin anti-Xa assessment in patients transitioned from DOAC to UFH. 27 28 As direct factor Xa assays have only a recorded lower limit of detection, results are reported as less than the lower limit of detection rather than zero. Therefore, despite the use of DOAC stop, some may argue that it is possible to have some influence on the heparin anti-Xa assay. This can be particularly true at least for some reagents since the DOAC-specific assays are set up on a dilution of patient plasma, and the Heparin anti-Xa is set up on neat plasma, which allows for even low levels of DOAC to have a magnified effect on heparin anti-Xa results.

**Fig. 2:**
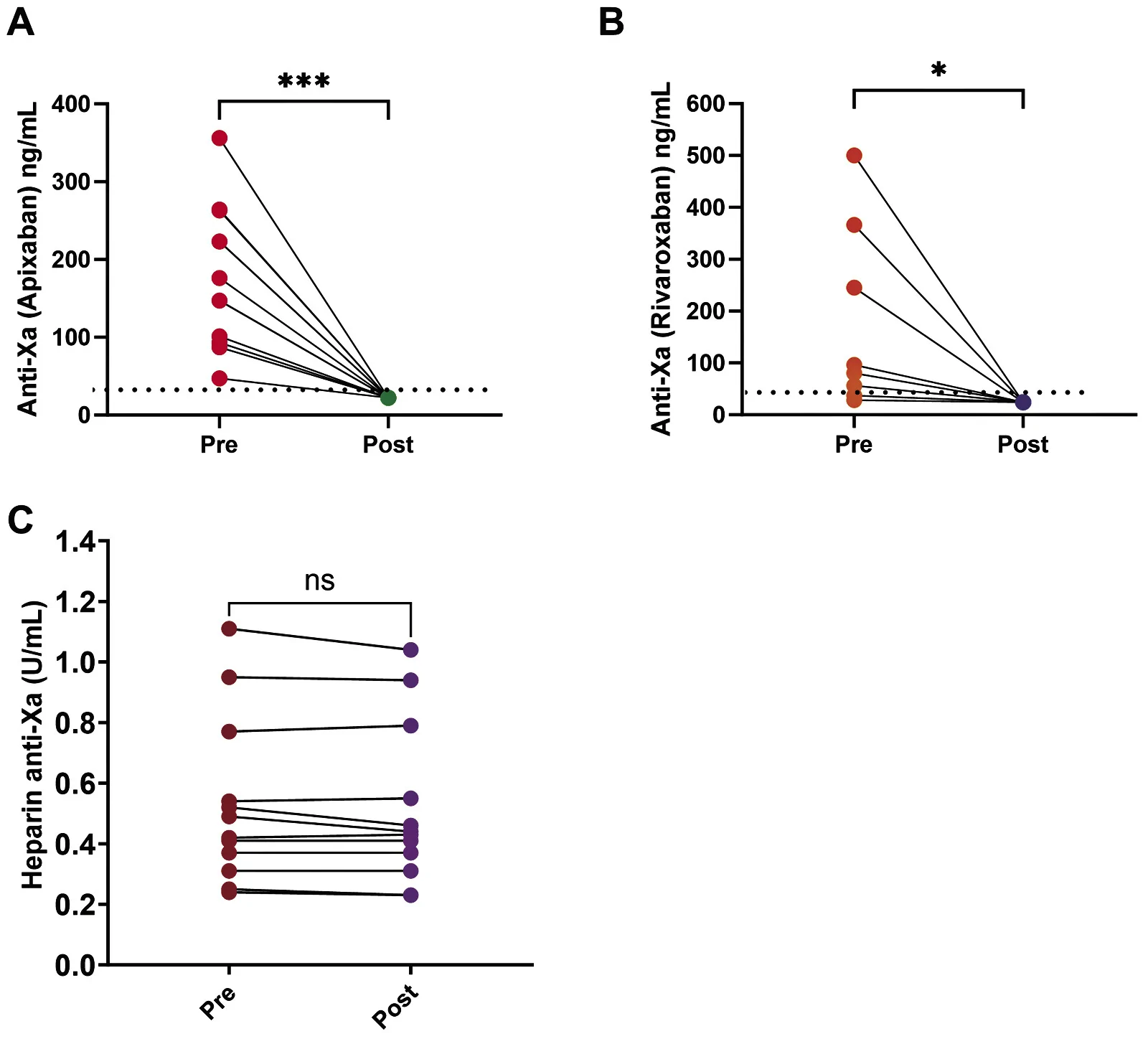
DOAC stop can eliminate the effects of apixaban ( **A** ) or rivaroxaban ( **B** ) in samples taken from patients, while DOAC-stop has no effect on ( **C** ) heparin anti-Xa levels when measured with unfractionated specific calibrators. Pre, prior to treating the samples with DOAC-stop; post, supernatant taken from the samples following treatment with DOAC-stop.

## Effect of DOAC Stop on Direct FXa Inhibitors/UFH Anti-Xa on Patient Plasma

It is important to know that when samples obtained from patients on UFH are treated with DOAC-stop, this does not affect the heparin anti-Xa assay, as shown in Fig. 2A .

Three examples ( Table 3 ) demonstrate that direct factor Xa inhibitors at therapeutic doses can produce supra-therapeutic heparin anti-Xa levels despite using drug-specific calibrators for heparin anti-Xa. In response to the previously mentioned concern that samples may have residual DOAC anti-factor Xa activity present despite the use of DOAC-stop, 28 we show that the effect on the heparin anti-Xa assay with a drug-specific calibrator is minimal ( Table 3 ).

**Table 3:** Direct factor Xa inhibitors can produce apparent supra-therapeutic heparin anti-Xa levels despite using drug-specific calibrators for heparin anti-Xa. DOAC-stop can eliminate this effect

	Pre-DOAC stop	Post-DOAC stop
Patient 1		
Rivaroxaban level (ng/mL)	551	<25
UFH anti-Xa (U/mL)	>2.00	<0.10
LMWH anti-Xa (U/mL)	>2.00	<0.10
Patient 2		
Rivaroxaban level (ng/mL)	216	<25
UFH anti-Xa (U/mL)	>2.00	<0.10
LMWH anti-Xa (U/mL)	>2.00	<0.10
Patient 3		
Apixaban (ng/mL)	360	<23
UFH anti-Xa (U/mL)	>2.00	<0.10
LMWH anti-Xa (U/mL)	>2.00	<0.10

## Practical Guidance on Managing Patient Transitioning from Direct Oral Anticoagulants to UFH

In a patient recently treated with dabigatran and starting UFH, it is straightforward to monitor UFH using heparin anti-Xa with specific calibrators since this assay is not affected by dabigatran. ( Fig. 3 ) It is generally recommended not to use a bolus dose of UFH since this may increase the risk of bleeding due to the combined effect of anticoagulants in a critically unwell patient. Since many laboratories still use APTT or ACT in situations where high-dose UFH is required, it is important to consider that dabigatran prolongs the APTT and ACT to variable degrees depending on time since last dose of dabigatran, renal function, and other PK factors.

**Fig. 3:**
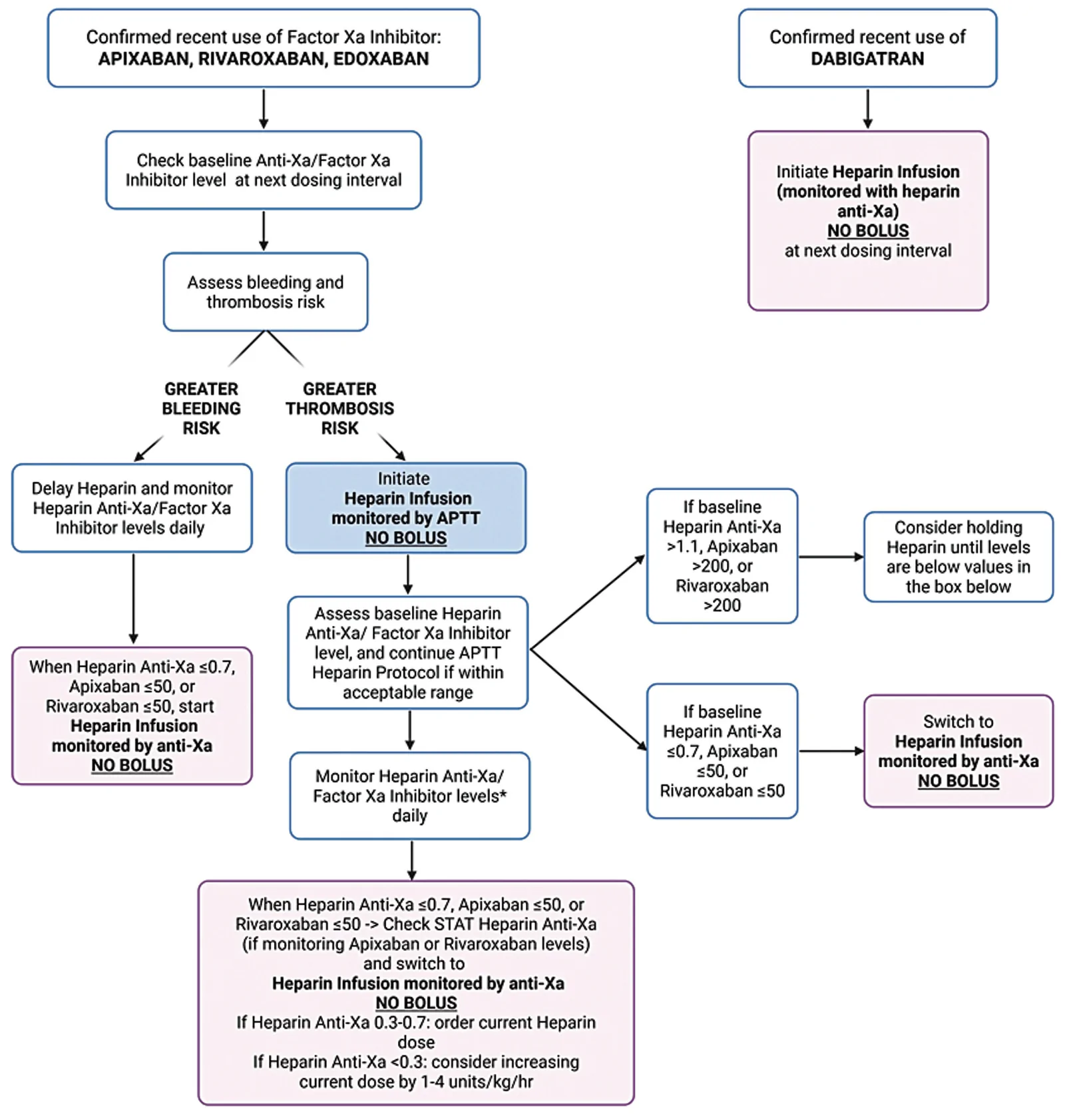
Suggested algorithm to manage patients transitioning from DOAC to UFH. Source: Created in BioRender. Arachchillage, D. (2025) https://BioRender.com/ia6is6k

In a patient who has been on a direct factor Xa inhibitor such as rivaroxaban, apixaban, or edoxaban, as discussed above, these drugs affect the heparin anti-Xa assay and lead to overestimation of the heparin anticoagulant effect. If it is confirmed or suspected that the patient has been on a direct factor Xa inhibitor, we suggest performing drug-specific anti-Xa assays for both the direct factor Xa inhibitor and heparin prior to starting UFH, while assessing the bleeding and thrombotic risks of the patient. Whether to start UFH is dependent on the anti-Xa levels and whether the patient is at greater risk of bleeding or thrombosis, although the general recommendation is to start UFH when the patient is due for their next dose of DOAC. 33 If the patient is at greater risk of bleeding, we suggest delaying the start of UFH until the apparent heparin anti-Xa level is ≤ 0.7 IU/mL and the direct factor Xa level for the specific drug is < 50 ng/mL. Once the above requirements are fulfilled, UFH infusion can be started without a bolus dose, the heparin anti-Xa level measured after 4 to 6 hours, and the UFH infusion adjusted accordingly. If the patient is at greater risk of thrombosis, we suggest initiating UFH without a bolus dose and monitoring UFH using APTT, since the effect of direct factor Xa inhibitors on APTT is small, especially with apixaban and exdoxaban. It is advisable to assess the baseline APTT to make sure there is no unexpected prolongation of APTT, which would then overestimate the UFH anticoagulant effect. However, if the patient's heparin anti-Xa level is > 1.1 IU/mL and drug-specific DOAC > 200 ng/mL, holding the initiation of UFH infusion should be considered due to a higher risk of bleeding, and daily assessment of heparin anti-Xa and drug-specific anti-Xa is suggested until they reach an anti-Xa level ≤ 0.7 IU/mL for UFH and < 50 ng/mL for drug-specific direct factor Xa level. Once these levels are achieved, it is suggested to monitor UFH with anti-Xa and adjust the UFH infusion accordingly ( Fig. 3 ).

Although direct factor Xa inhibitors interfere with the LMWH anti-Xa as well, monitoring of LMWH is seldom required. If it is necessary to assess the LMWH anti-Xa levels, results should be interpreted with caution, taking into account the effect of recent direct factor Xa inhibitor effect of the anti-Xa level.

In conclusion, DOAC are the mainstay of anticoagulation for many indications at present, but UFH remains the anticoagulant of choice for those critically unwell and with organ dysfunction. When a patient has been on DOAC and transitions to UFH, the effects of DOAC on assays that are used to monitor UFH should be taken into account, and UFH monitoring adjusted accordingly to avoid the increased risk of thrombosis or bleeding.
